# A new highly sensitive real-time quantitative-PCR method for detection of *BCR-ABL1* to monitor minimal residual disease in chronic myeloid leukemia after discontinuation of imatinib

**DOI:** 10.1371/journal.pone.0207170

**Published:** 2019-03-05

**Authors:** Hiroaki Kitamura, Yoko Tabe, Tomohiko Ai, Koji Tsuchiya, Maiko Yuri, Shigeki Misawa, Takashi Horii, Atsushi Kawaguchi, Akimichi Ohsaka, Shinya Kimura

**Affiliations:** 1 Division of Hematology, Respiratory Medicine and Oncology, Department of Internal Medicine, Faculty of Medicine, Saga University, Saga, Japan; 2 Department of Next Generation Hematology Laboratory Medicine, Juntendo University Graduate School of Medicine, Bunkyo-ku, Tokyo, Japan; 3 Department of Clinical Laboratory Medicine, Juntendo University Graduate School of Medicine, Bunkyo-ku, Tokyo, Japan; 4 Department of Clinical Laboratory, Juntendo University Hospital, Bunkyo-ku, Tokyo, Japan; 5 Education and Research Center for Community Medicine, Faculty of Medicine, Saga University, Saga, Japan; University of British Columbia, CANADA

## Abstract

Tyrosine kinase inhibitors (TKIs) targeting the *BCR-ABL1* fusion protein, encoded by the Philadelphia chromosome, have drastically improved the outcomes for patients with chronic myeloid leukemia (CML). Although several real-time quantitative polymerase chain reaction (RQ-PCR) kits for the detection of *BCR-ABL1* transcripts are commercially available, their accuracy and efficiency in laboratory practice require reevaluation. We have developed a new in-house RQ-PCR method to detect minimal residual disease (MRD) in CML cases. MRD was analyzed in 102 patients with CML from the DOMEST study, a clinical trial to study the rationale for imatinib mesylate discontinuation in Japan. The *BCR-ABL1*/*ABL1* ratio was evaluated using the international standard (IS) ratio, where IS < 0.1% was defined as a major molecular response. At enrollment, *BCR-ABL1* transcripts were undetectable in all samples using a widely-applied RQ-PCR method performed in the commercial laboratory, BML (BML Inc., Tokyo, Japan); however, the in-house method detected the *BCR-ABL1* transcripts in five samples (5%) (mean IS ratio: 0.0062 ± 0.0010%). After discontinuation of imatinib, *BCR-ABL1* transcripts were detected using the in-house RQ-PCR in 21 patients (21%) that were not positive using the BML method. Nineteen samples were also tested using a commercially available RQ-PCR assay kit with a detection limit of IS ratio, 0.0032 (ODK-1201, Otsuka Pharmaceutical Co., Tokyo, Japan). This method detected low levels of *BCR-ABL1* transcripts in 14 samples (74%), but scored negative for five samples (26%) that were positive using the in-house method. From the perspective of the in-house RQ-PCR method, number of patients confirmed loss of MMR was 4. These data suggest that our new in-house RQ-PCR method is effective for monitoring MRD in CML.

## Introduction

Chronic myeloid leukemia (CML) is a disease that arises in hematopoietic stem cells and is caused by a reciprocal translocation between chromosomes 9 and 22 (t(9;22)(q34;q11.2)), referred to as the Philadelphia chromosome, which generates *BCR-ABL1* fusion transcripts. The *BCR-ABL1* protein constitutively activates tyrosine kinase (TK) [1] that causes unregulated proliferation of abnormal blood cells, and consequently interrupts normal hematopoiesis. Theoretically, TK inhibition was expected to be an effective cure for CML, and imatinib, which competitively inhibits phosphorylation of *BCR-ABL1*, was developed in 2001 and is used as a frontline TK inhibitor (TKI) [2–5]. Currently, according to the European Society of Medical Oncology (ESMO) Clinical Practice Guideline (2017), three commercially available TKIs, imatinib, dasatinib, and nilotinib, can be used for the CML therapy with no significant difference in survival rate [6].

To monitor the response to treatment with TKI, several assessment methods have been employed as follows: (1) complete hematologic response, determined by examination of complete blood cell counts and differentiated by flow cytometry; (2) complete cytogenetic response, evaluated using bone marrow aspirate and biopsy samples; and (3) molecular response (MR) examined by real-time quantitative-PCR (RQ-PCR) [7]. Of these, MR detection by RQ-PCR is the most sensitive method to monitor minimal residual disease (MRD); however, RQ-PCR protocols vary among laboratories, potentially leading to inconsistencies in patient treatment. Therefore, the European Leukemia Network (ELN) and National Comprehensive Cancer Network (NCCN) have recommended monitoring *BCR-ABL1* mRNA levels by RQ-PCR using international standards (IS) [8–10]. The International Randomized Study of Interferon versus STI571 (IRIS) proposed that log reduction of *BCR-ABL1*^IS^ (IS ratio) during therapy, compared with baseline IS ratio at diagnosis (*BCR-ABL1*^IS^, 100%), should be evaluated to monitor MRD. Initially, major molecular response (MMR), defined as *BCR-ABL1*^IS^ ≤ 0.1% (MR: 3.0; 3 log reduction) was considered adequate [11]. Subsequently, deeper molecular responses (DMRs) were determined to be desirable. DMRs are defined as *BCR-ABL1*^IS^ ≤ 0.01% (MR: 4.0; 4 log reduction), *BCR-ABL1*^IS^ ≤ 0.0032% (MR: 4.5; 4.5 log reduction), and *BCR-ABL1*^IS^ ≤ 0.001% (MR: 5.0; 5 log reduction) [12].

Long-term treatment with TKIs can cause considerable adverse effects, including gastrointestinal damage, fluid retention, bone marrow suppression, liver injury, cardiovascular events, and kidney injury [13]. Even more seriously, some patients develop resistance to imatinib [14]. In some cases, imatinib must be discontinued or replaced with a different TKI, such as bosutinib, because of such problems. Consequently, several clinical trials have been conducted to investigate whether TKIs can be ceased after DMR is achieved. Mahon *et al*. reported that approximately 40% of patients with CML remained in complete molecular response (CMR) for at least 2 years after discontinuation of imatinib [15]. Stop studies of second-generation TKIs (dasatinib and nilotinib) showed that approximately 50% of patients achieved, and remained in, DMR following TKI cessation [16, 17]. As DMR is an emerging goal in CML and necessary for entry into treatment discontinuation studies [15, 18, 19], RQ-PCR assays with inadequate sensitivity could fail to detect low level *BCR-ABL1* fusion transcripts, leading to inappropriate or premature treatment cessation attempts. Therefore, well defined guidelines have been developed to ensure adequate sensitivity levels are achieved, down to MR4.0 or MR4.5 [20]. The World Health Organization International Genetic Reference Panel for the quantitation of *BCR-ABL1* mRNA (World Health Organization document, World Health Organization/BS/09.2106) has been distributed to manufacturers to generate secondary reference materials [21], and commercial kits are now available from several manufacturers [22].

Recently, we developed a new in-house RQ-PCR method and determined its sensitivity as 0.0033% using synthetic ARQ IS Calibrator Panels; this level of sensitivity is sufficient to detect MRD [23]. In this study, we evaluated the ability of this in-house RQ-PCR method to detect low level *BCR-ABL1* fusion transcripts using samples obtained in the ongoing Delightedly Overcome CML Expert Stop TKI (DOMEST) clinical trial to evaluate the rationale for cessation of imatinib [24].

## Materials and methods

### Study design

This study was performed as a part of the DOMEST clinical trial, which was conducted to elucidate the rationale for imatinib discontinuation in Japan [24]. The enrollment criteria were (1) 15 years of age or older, (2) diagnosed with CML in chronic phase and receiving imatinib therapy, and (3) maintained DMR for longer than 2 years (MR4.0 or MR4.0 equivalent), as determined by transcription-mediated amplification, reverse transcriptase-polymerase chain reaction (RT-PCR), or real-time quantitative polymerase chain reaction (RQ-PCR). Other inclusion criteria were a WHO performance status score of 0–2 and absence of severe dysfunction of primary organs. Previous therapies additional to imatinib were permitted. Patients with additional chromosomal abnormalities and those with a positive RQ-PCR result using the method applied by BML (BML Inc., Tokyo, Japan) at the time of registration were excluded. The study was approved by the ethics committees of Saga University Graduate School of Medicine and Juntendo University Graduate School of Medicine. All participants provided written informed consent for their samples and data from their medical records to be used for research.

In the DOMEST study, RQ-PCR was performed every month for the first year and every 3 months for the second year by the BML method [16, 25]; molecular recurrence was defined as *BCR-ABL1* detected by two successive tests, or by loss of MR3.0 in one test by the BML method. Residual total RNA samples were subsequently used for measurement using the in-house RQ-PCR method if samples were available. Although we confirmed that e13a2 (b2a2) and e14a2 (b3a2) can be quantified with the in-house method, sequencing is necessary to differentiate transcripts. Samples were measured with the in-house method at the time of the registration and when recurrence was confirmed by the BML method. The major *BCR-ABL1* mRNA assay kit, ODK-1201 (Otsuka Pharmaceutical Co., Japan), which also uses the RQ-PCR technique, was used to test available samples showing discrepant results between the in-house and BML methods for comparison [26]. When the DOMEST study was designed, the in-house method was not approved for clinical use, and ODK-1201 were not yet available. Therefore, these two methods did not contribute to the clinical decision for the DOMEST study.

### RNA extraction and cDNA synthesis

Total RNA was extracted from 7 mL peripheral blood in EDTA tubes using a QIAamp RNA Blood Mini Kit (Qiagen, Hilden, Germany). RNA was quantified by Nanodrop spectrophotometry (ND 2000-NanoDrop 3.2.1, Thermo Scientific, Waltham, USA). Transcriptor Universal cDNA Master reverse transcriptase (Roche Diagnostics, Mannheim, Germany) was used for cDNA synthesis, using 1 μg total RNA.

### Quantitative real-time PCR

cDNA was amplified by 55 cycles of RT-PCR in a final reaction volume of 20 μL using the LightCycler 2.0 (Roche Diagnostics, Mannheim, Germany) and LightCycler TaqMan Master, in accordance with the manufacturer’s instructions. *ABL1* was used as the control gene. The primers and probes used were as follows: *BCR-ABL1* forward primer, 5'-TGACCAACTCGTGTGTGAAACTC-3', reverse primer, 5'-CACTCAGACCCTGAGGCTCAA-3', and probe, 5'- CCCTTCAGCGGCCAGTAGCATCTGA-3'; *ABL1* forward primer, 5'-CGAAGGGAGGGTGTACCATTA-3', reverse primer, 5'- CAACTCGGCCAGGGTGTT-3', and probe, 5'-CTTCTGATGGCAAGCTCTACGTCTCCTCC-3'. Sequences were obtained from GenBank (Accession Nos. X02596 for *BCR* and X16416 for *ABL1*). Probes contained the fluorescent reporter dye, 6-carboxyfluorescein (FAM), at the 5'-end and the fluorescent quencher dye, Black Hole Quencher (BHQ), at the 3'-end. Results are reported as *BCR-ABL1*/*ABL1* ratios (%).

### RNA standards for the RQ-PCR assay

An *in vitro* transcribed RNA from the *BCR-ABL1* gene of the K562 cell line was used to determine the lower detection limit of the assay. A region of 188 bp, including the *BCR-ABL1* breakpoint, was amplified by PCR using the primers described above. The product was purified using the QIAquick PCR Purification Kit (Qiagen, Hilden, Germany) and then ligated to the pGEM-T vector (Promega, Madison, USA). The recombinant plasmid was transformed to the DH5α *Escherichia coli* strain (Promega), and the cloned plasmid was extracted using a QIAprep Spin Miniprep Kit (Qiagen). The orientation of the DNA insert was confirmed by sequencing. *In vitro* transcription was performed using either the RiboMAX Large Scale RNA Production System or the T7 RiboMAX Express Large Scale RNA Production System (Promega), depending on the direction of inserts, as determined by sequencing. Transcribed RNA was purified using the RNeasy Mini Kit (Qiagen), and the amount of RNA was quantified using the Agilent RNA 6000 Nano Assay (Agilent Technology, California, USA).

The RNA copy number (/μl) was calculated using the following equation:

$$\underline{C\;x\;A}$$

329xL

where C is the concentration of RNA (g/μl), assessed using the Agilent 2100 Bioanalyzer; A is Avogadro’s constant (6.0 × 10^23^ copies/mol); L is the length of synthetic RNA (nucleotides); and 329 is an approximation of the molecular weight of a nucleotide (g/mol).

### Determination of a laboratory-specific correlation parameter (CP) and data analyses

The World Health Organization (WHO) established an international genetic reference panel for quantification of *BCR-ABL1* fusion transcripts by RQ-PCR, which contains four different ratios (10%, 1%, 0.1%, and 0.01%) using the *BCR-ABL1*-positive cell line, K562, diluted in the *BCR-ABL1*-negative cell line, HL60 [21]. Four level Armored RNA Quant (ARQ) (Asuragen, Inc., Austin, TX, USA) secondary reference panels were manufactured based on the WHO primary standards [22]. Laboratory-specific CP equivalent conversion factor values were calculated for use with the ARQ IS Calibrator Panels.

**Fig 1:**
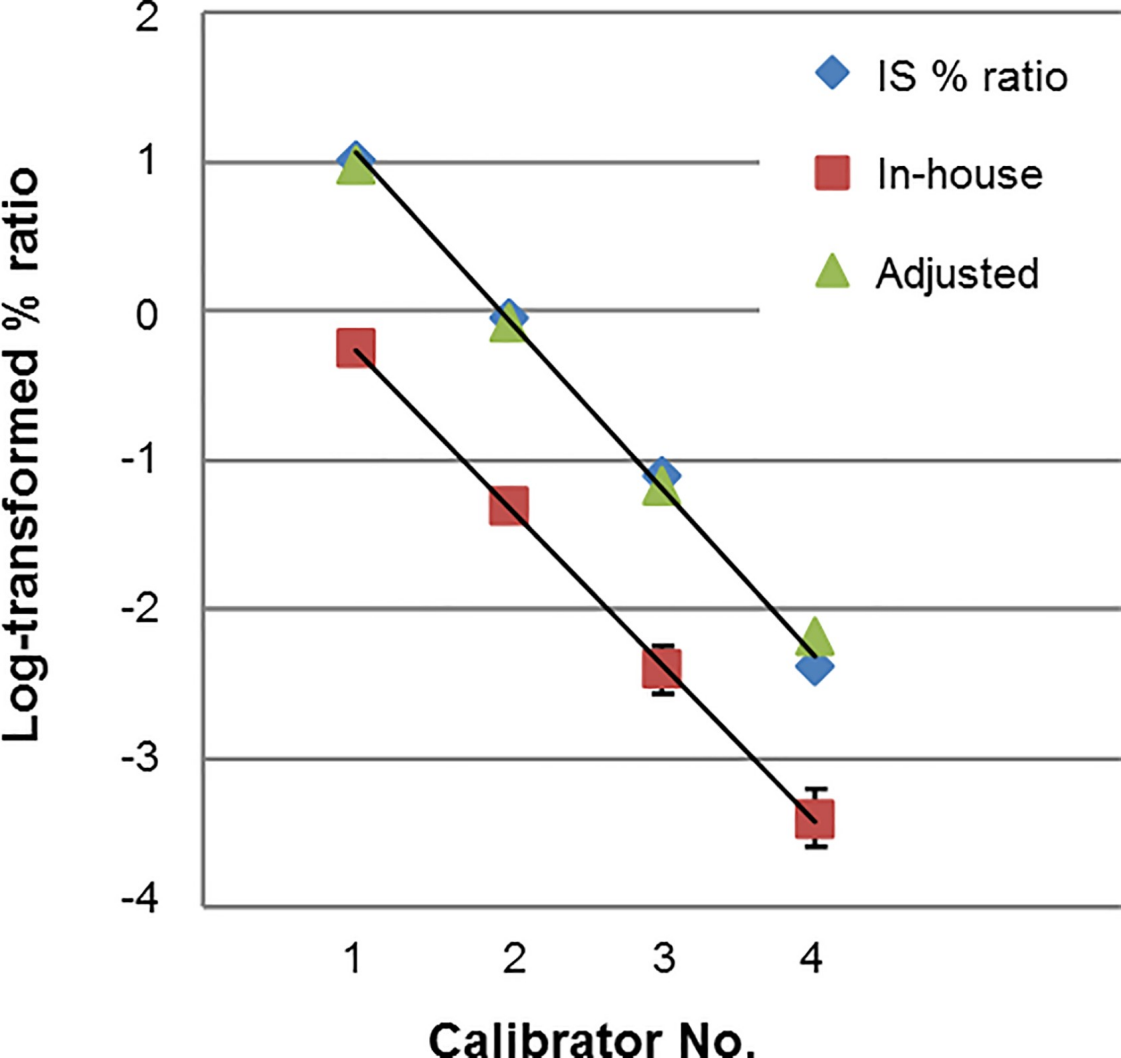
Evaluation of the accuracy of the in-house method in comparison to WHO reference panel IS, International scale.

Following the previous calibration method using the ARQ IS Calibrator Panel™ [23], the CP of the in-house RQ-PCR method was redetermined using another ARQ IS Calibrator Panel™ lot containing four calibrators: IS 4.1%, 0.37%, 0.027%, and 0.0033%. S1 Table shows the ARQ IS Calibrator Panel in six independent runs using the in-house RQ-PCR to determine the CP. Fig 1 shows the plot of measured calibrators using the in-house method. The estimated CP value for this study was 18.39. The *BCR-ABL1* mRNA ratio of standard material RNAs supplied by the panel was quantified using the local method, and 95% limits of agreement (LOA) were calculated. Values outside of the 95% LOA were omitted, and the CP was calculated by dividing the measured value by the expected value.

### Sequencing analysis

*BCR-ABL1* PCR products were separated and purified using agarose gel electrophoresis and a QIAquick Gel Extraction Kit (Qiagen). Cycle sequencing was performed using a BigDye Terminator v1.1 Cycle Sequencing Kit (Applied Biosystems, Foster City, USA). Cycle sequencing products were purified using a BigDye Xterminator Purification Kit (Applied Biosystems, Foster City, USA) before being run on an automated ABI 3500 genetic analyzer (Applied Biosystems), and sequences were analyzed using Sequencing Analysis software ver.6.

### Statistical analysis

Correlation analyses were performed using the Pearson’s correlation coefficient. *BCR-ABL1* undetectable rate represented as survival curve was estimated by the Kaplan-Meier method. These were compared paired groups (BML method and in-house method) by the hazard ratio estimated through the Cox proportional hazards model with the robust sandwich variance estimator. We considered *p* values < 0.05 to indicate significant differences. Statistical analysis was performed using statistical computing R.

## Results

### Background of patients

Between January 2014 and May 2015, a total of 110 patients were enrolled for the DOMEST study; 104 of them were evaluated in this study. Among these patients, 102 were confirmed as having DMR (MR4.0) status, defined as “*BCR-ABL1* transcript levels below the detection limit of the widely-used BML method.” After MR4.0 (Log4) was confirmed, imatinib was ceased. The other two patients were excluded from this study because *BCR-ABL1* transcripts were detected at enrollment, and dasatinib was started.

### Comparison of *BCR-ABL1* mRNA levels measured by the BML and in-house RQ-PCR methods during follow-up of imatinib discontinuation

**Fig 2:**
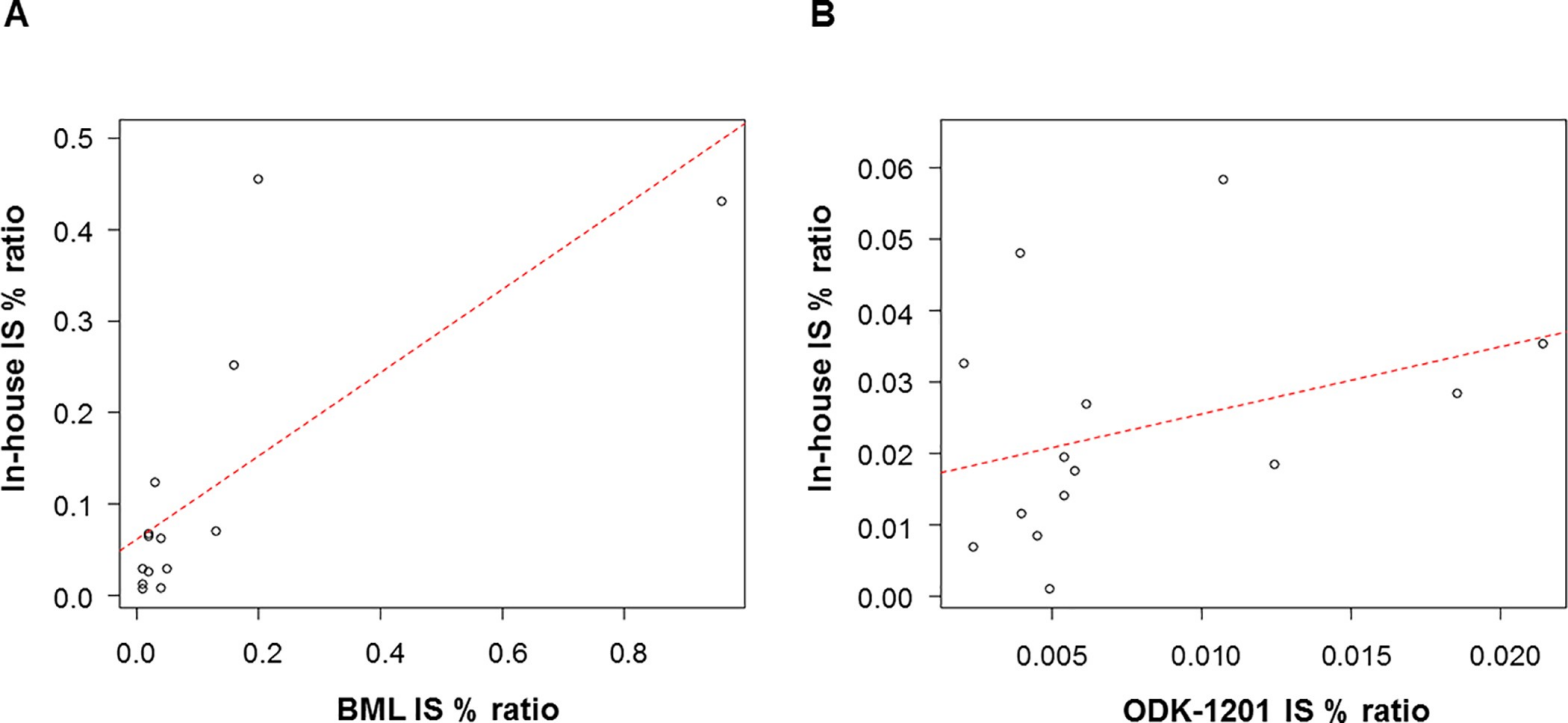
Correlation of IS% *BCR-ABL1*/*ABL1* among the three methods. **Correlations between the BML and in-house methods (A) and the in-house and ODK-1201 methods (B).** IS, International scale.

In the DOMEST study, total number of measurement by the BML method was 1303. Using the in-house RQ-PCR method, we evaluated available 233 samples of the 1303 samples at the begging of the study and when *BCR-ABL1* transcripts were detected by the BML method (i.e., molecular recurrence). Unexpectedly, despite confirmation of DMR using the BML method in all enrolled cases, *BCR-ABL1* transcripts were detected by the in-house RQ-PCR method in 5 of 102 patients (5%) at the beginning of the DOMEST study. The IS ratios detected using the in-house method in these five cases are presented in S2 Table. The sequences of the PCR amplicons were confirmed by Sanger sequencing (S1 Fig); however, in the DOMEST study, these five patients remained in MR4.0, as determined by the BML method, throughout the study. Subsequently, *BCR-ABL1* fusion transcripts were detected in 15 cases (15%) by the BML method and the in-house method at the same time points, at an average ± standard deviation of 2.47 ± 2.13 months after cessation of imatinib (concordant cases, Table 1). In one case (patient #15), the fusion transcript level was < 0.01% by the BML method. Fig 2A shows the correlation between the BML and the in-house methods. IS % *BCR-ABL1/ABL1* measured by these two methods were plotted (except patient #15), and it was fitted with a linear regression method, yielding a slope of 0.75 (*p* = 0.0022). Concordance rates between the two methods for IS % ratio >0.1, ≤0.1 - >0.01 and ≤0.01 were 75.0% (3/4), 71.4% (5/7) and 50.0% (2/4), respectively. In these recurred cases, TKI therapies were restarted in the DOMEST study (Table 1).

**Table 1:** Cases with concordant results for detection of *BCR-ABL1* fusion transcripts using the BML and in-house methods.

Patient #	Timing of detection	IS % ratio		TKI type after recurrence
	(month)	BML method	In-house method	
6	9	0.01	0.0289	Dasatinib
7	2	0.04	0.0621	Imatinib
8	1	0.02	0.0259	Dasatinib
9	3	0.20	0.4548	Dasatinib
10	1	0.02	0.0648	Dasatinib
11	1	0.96	0.4306	Dasatinib
12	2	0.13	0.0700	Imatinib
13	1	0.01	0.0121	Dasatinib
14	3	0.01	0.0069	Dasatinib
15	1	<0.01	0.0093	Imatinib
16	1	0.04	0.0081	Dasatinib
17	3	0.16	0.2517	Dasatinib
18	2	0.05	0.0292	Imatinib
19	2	0.02	0.0672	Imatinib
20	5	0.03	0.1236	Dasatinib

IS, International scale.

Timing of detection: time point when *BCR-ABL1* fusion transcripts were detected.

By contrast, the results were discordant between the BML and in-house methods in 21 cases (21%) (Table 2). The in-house method detected *BCR-ABL1* fusion transcripts at an average (range) of 2.4 (1–13) months earlier than the BML method. TKI therapies were restarted in these cases (Table 2). Of these 21 cases, 19 available samples were also tested using another RQ-PCR assay kit (ODK-1201; Otsuka Pharmaceutical, Japan), and the results were compared with those from the in-house method. As shown in Table 2, the ODK-1201 method detected a low level (IS < 0.01%) of *BCR-ABL1* fusion transcripts in 14 samples (74%), while they were detected in all 19 cases using the in-house method. However, the correlation coefficient of the IS% ratios detected by the in-house method and ODK-1201 (except patient #21, 22, 23, 30, 32, 38 and 41) was weak (r = 0.35, *p* = 0.22, Fig 2B). Concordance rates between the two methods for IS % ratio >0.1, ≤0.1 - >0.01, ≤0.01 - >0.001 and ≤0.001 evaluated by the in-house method were 0.0% (0/0), 31.0% (4/13), 40.0% (2/5) and 0.0% (0/1), respectively.

**Table 2:** Cases with discordant results for detection of *BCR-ABL1* fusion transcripts using the BML and in-house methods.

Patient #	Timing of detection (month)		IS % ratio		TKI type after recurrence
	BML method	In-house method	In-house method	ODK-1201	
21	15	12	0.0201	ND	Dasatinib
22	18	5	0.0041	ND	Dasatinib
23	1	0	0.0081	NA	Dasatinib
24	11	4	0.0184	0.0124	Dasatinib
25	5	3	0.0584	0.0107	Dasatinib
26	1	0	0.0326	0.0021	Dasatinib
27	4	2	0.0269	0.0061	Dasatinib
28	15	12	0.0141	0.0054	Dasatinib
29	2	1	0.0115	0.0040	Dasatinib
30	1	0	0.0127	NA	Imatinib
31	3	2	0.0284	0.0186	Dasatinib
32	1	0	0.0120	ND	Imatinib
33	5	4	0.0353	0.0214	Dasatinib
34	4	3	0.0481	0.0039	Dasatinib
35	2	0	0.0069	0.0024	Imatinib
36	1	0	0.0194	0.0054	Dasatinib
37	6	3	0.0010	0.0049	Dasatinib
38	3	0	0.0082	ND	Dasatinib
39	1	0	0.0175	0.0058	Imatinib
40	1	0	0.0084	0.0045	Dasatinib
41	3	1	0.0019	ND	Dasatinib

IS, International scale; ND, not detected; NA, not applicable.

Timing of detection: time point when *BCR-ABL1* fusion transcripts were detected.

*BCR-ABL1* fusion transcripts were detected by the in-house method in all samples positive by the BML method. In the remaining 61 cases, *BCR-ABL1* fusion transcripts were not detected using either the in-house or BML methods throughout the study.

### Comparison of distribution of IS % ratio and timing of detection of *BCR-ABL1* between the BML and in-house methods

**Fig 3:**
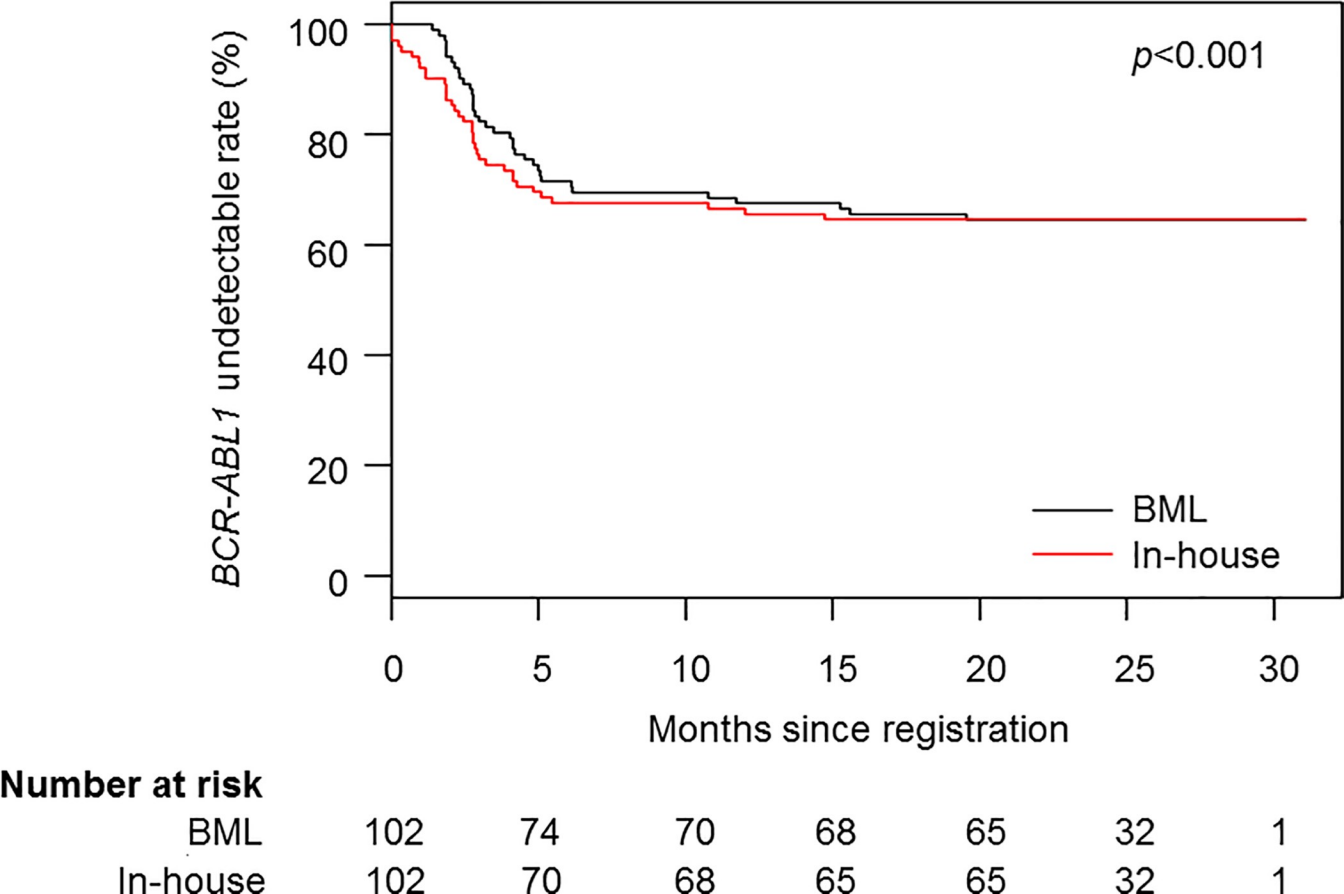
Kaplan-Meier curves of *BCR-ABL1* undetectable rate comparing with the BML method and in-house method.

Table 3 summarizes the comparison of distribution of the *BCR-ABL1*/*ABL1* ratio according the IS at the time of the detection of *BCR-ABL1* between the BML method and in-house methods. The number of patients confirmed loss of MMR was 4 by the in-house method. Fig 3 shows *BCR-ABL1* undetectable rate comparing with the BML method and in-house methods. The in-house method detected significantly lower levels of *BCR-ABL1* fusion transcripts in comparison to the BML method (HR: 1.07, 95% CI: 1.03–1.11, *p*<0.001).

**Table 3:** Comparison of distribution of IS % *BCR-ABL1*/*ABL1* between the BML and in-house methods.

IS % ratio	BML method (n = 102)	In-house method (n = 102)
>0.1	8	4
≤0.1->0.01	22	22
≤0.01-Detection limit	6	15
Undectable *BCR-ABL1*	66	61

IS, International scale.

### Restart of TKI treatment after molecular recurrence

In the current study, 36 patients with confirmed molecular recurrence were included. However, one patient was excluded because of missing data according to the report of the DOMEST study. Therefore, the authors analyzed 35 patients with confirmed molecular recurrence after cessation of imatinib treatment. Of the 35 patients, 26 patients were retreated with dasatinib and 9 patients were retreated with imatinib on their wish. Moreover, 21 (80.8%) and 25 (96.2%) of the 26 patients treated with dasatinib regained DMR at 6 and 12 months after restarting, respectively. In the DOMEST study, only patients treated with dasatinib were analyzed as a purpose of the secondary endpoint [24].

## Discussion

Detection of low levels of *BCR-ABL1* fusion transcripts to monitor MRD must be performed quickly, efficiently, and at a reasonable cost [27]. Currently, several commercial kits using IS are available [22, 28]; however, some of these kits are designed to test large-scale samples in commercial laboratories, and some kits need specific reagents and analyzers whose costs are significantly expensive [29]. Therefore, we developed an in-house RQ-PCR method that is sufficiently accurate and more flexible compared to those currently in use [23]. Our in-house method can be performed using any RQ-PCR reagents and analyzers as long as calibration can be made. In the present study, we evaluated the relevance and accuracy of this in-house RQ-PCR method using samples obtained for the DOMEST trial. Our data demonstrate that the in-house method is sufficiently sensitive to detect MRD and recurrence, relative to the widely-used BML method and the recently developed ODK-1201 commercial kit.

In the DOMEST trial, clinical decisions were made based on the monitoring of *BCR-ABL1* fusion transcripts measured using the BML method (detection limit: IS 0.01%) (BML Inc.) [25]. The *BCR-ABL1* mRNA quantification results obtained using the in-house RQ-PCR agreed with those generated using the BML method in 15 cases (Table 2). By contrast, the in-house method detected MRD earlier than the BML method in 21.0% of cases (Table 3). Although both the in-house and ODK-1201 methods can identify at least a 4.5 log reduction in the IS ratio [26], the IS ratios measured in this study were somewhat discordant between the two methods. In five samples, the IS ratios were below the initially claimed detection limit (IS ratio < 0.0007%) of the ODK-1201 method, whereas using the in-house method they had a mean IS ratio of 0.0094 ± 0.00754%. In 13 cases, the IS ratios determined using the ODK-1201 method were lower than those using the in-house method. This discordance might be due to RNA degradation since measurements could not all be performed at exactly the same time. Alternatively, it could be due to the relatively large variability in the detection of very low copy number transcripts, which is unavoidable using current technology. In the initial validation study of ODK-1201, the IS% *BCR-ABL1/ABL1* calibration was performed using WHO standard product [NIBSC 09/138, the National Institute for Biological Standards and Control (NIBSC), UK] that is different from the one used in this study. Although the detection limit of ODK-1201 was claimed to be 00007%, the IS% *BCR-ABL1/ABL1* obtained from the 154 samples from CML patients fell into between 0.0014% and 144.6867%. The IS% below the detection limit obtained from CML patients (70 samples), 21 non-CML patients and 25 healthy subjects were represented as 0.0001% [26]. This may be problematic since one cannot tell the absolute IS% values if those are below the detection limit. Also, the status of the CML patients with the negative results is unclear. In fact, the same group later reevaluated the ODK-1201, and the detection level was re-claimed to be IS% 0.0032 [30], which indicates that RT-qPCR measurement of very small number of RNA molecules may not be very accurate. Indeed, we compared the in-house method and ODK-1201 using the samples whose *BCR-ABL1* transcripts were supposed to be very low, and we could compare only 19 samples due to sample availability. Taking these facts together, the discordance between these two methods may not be surprised when such small amount of RNA molecule(s) are measured.

In recent clinical trials to evaluate the rationale for TKI cessation, the sensitivity of assays used for detection of *BCR-ABL1* fusion transcripts has been claimed as 5 logs [12, 31]; however, there are reasons to be skeptical about the accuracy of measurements of such extremely low amounts of mRNA. Even a subtle pipetting error can easily lead to an enormous difference. In addition, despite using the IS, calculation and/or methods of determining conversion factors can significantly affect the results. According to the UKNEQAS (external quality assessment), the variability of results among participant laboratories was considerable, even after the introduction of an IS [32]. Therefore, the methodology used for the measurement of *BCR-ABL1* fusion transcripts requires further improvement.

This study has certain limitations. The clinical relevance of our new in-house RQ-PCR is uncertain since it has not been assessed in large-scale randomized clinical trials. Moreover, very long-term outcomes of imatinib therapy in CML have yet to be elucidated [33]. Current recommendations for the definition of MRs may be changed after accumulation of further data. Although we performed all experimental procedures with great care, the introduction of some errors caused by human factors cannot be completely excluded, as noted in a recent commentary [29].

In conclusion, our newly developed in-house RQ-PCR method with IS calibration was accurate and effective for detecting MRD in the context of an imatinib cessation study. The main advantages of this assay lie in the promptness with which results are obtained and its ease of use. Thus, this method could be advantageous for implementation in hospital laboratories, where small numbers of samples are tested.

## Supporting information

S1 Table Distribution of percent ratios determined by the ARQ IS calibrator panel. (DOCX)

S2 Table IS ratios generated using the in-house RQ-PCR method for samples where transcripts were not detected by the BML method at the beginning of the study. (DOCX)

S1 Fig PCR amplicon sequence data for patients detected by the in-house, but not the BML, methods at the beginning of the study. (TIF)
