# Supplementary material for: A new highly sensitive real-time quantitative-PCR method for detection of BCR-ABL1 to monitor minimal residual disease in chronic myeloid leukemia after discontinuation of imatinib
Source: PLoS One. 2019 Mar 5;14(3):e0207170. doi: 10.1371/journal.pone.0207170 (PMC6400442; doi:10.1371/journal.pone.0207170)
Supplement: S1 Table — (DOCX) [file pone.0207170.s001.docx]

**S1 Table. Distribution of percent ratios determined by the ARQ IS calibrator panel**

|  | % ratio | |  | Log-transformed % ratio | |
| --- | --- | --- | --- | --- | --- |
|  | IS | In-house |  | IS | In-house |
| Calibrator 1 | 10.3 | 0.56 |  | 1.013 | -0.254 |
|  | 10.3 | 0.61 |  | 1.013 | -0.218 |
|  | 10.3 | 0.57 |  | 1.013 | -0.242 |
|  | 10.3 | 0.61 |  | 1.013 | -0.218 |
|  | 10.3 | 0.54 |  | 1.013 | -0.266 |
|  | 10.3 | 0.525 |  | 1.013 | -0.280 |
| Calibrator 2 | 0.92 | 0.055 |  | -0.036 | -1.258 |
|  | 0.92 | 0.0456 |  | -0.036 | -1.341 |
|  | 0.92 | 0.044 |  | -0.036 | -1.353 |
|  | 0.92 | 0.041 |  | -0.036 | -1.382 |
|  | 0.92 | 0.0534 |  | -0.036 | -1.273 |
|  | 0.92 | 0.054 |  | -0.036 | -1.265 |
| Calibrator 3 | 0.078 | 0.0056 |  | -1.108 | -2.252 |
|  | 0.078 | 0.00345 |  | -1.108 | -2.462 |
|  | 0.078 | 0.0052 |  | -1.108 | -2.287 |
|  | 0.078 | 0.0024 |  | -1.108 | -2.612 |
|  | 0.078 | 0.00466 |  | -1.108 | -2.331 |
|  | 0.078 | 0.00233 |  | -1.108 | -2.632 |
| Calibrator 4 | 0.0041 | 0.00016 |  | -2.387 | -3.808 |
|  | 0.0041 | 0.00032 |  | -2.387 | -3.494 |
|  | 0.0041 | 0.00046 |  | -2.387 | -3.335 |
|  | 0.0041 | 0.00044 |  | -2.387 | -3.353 |
|  | 0.0041 | 0.00053 |  | -2.387 | -3.274 |
|  | 0.0041 | 0.00046 |  | -2.387 | -3.336 |

IS, International scale.
