# Supplementary material for: A new highly sensitive real-time quantitative-PCR method for detection of BCR-ABL1 to monitor minimal residual disease in chronic myeloid leukemia after discontinuation of imatinib
Source: PLoS One. 2019 Mar 5;14(3):e0207170. doi: 10.1371/journal.pone.0207170 (PMC6400442; doi:10.1371/journal.pone.0207170)
Supplement: S2 Table — (DOCX) [file pone.0207170.s002.docx]

**S2 Table. IS ratios generated using the in-house RQ-PCR method for samples where transcripts were not detected by the BML method at the beginning of the study.**

| Patient # | IS % ratio (In-house method) |
| --- | --- |
| 1 | 0.0077 |
| 2 | 0.0057 |
| 3 | 0.0067 |
| 4 | 0.0052 |
| 5 | 0.0059 |

IS, International scale.
