# Supplementary figures and images for: A new highly sensitive real-time quantitative-PCR method for detection of BCR-ABL1 to monitor minimal residual disease in chronic myeloid leukemia after discontinuation of imatinib
Source: PLoS One. 2019 Mar 5;14(3):e0207170. doi: 10.1371/journal.pone.0207170 (PMC6400442; doi:10.1371/journal.pone.0207170)

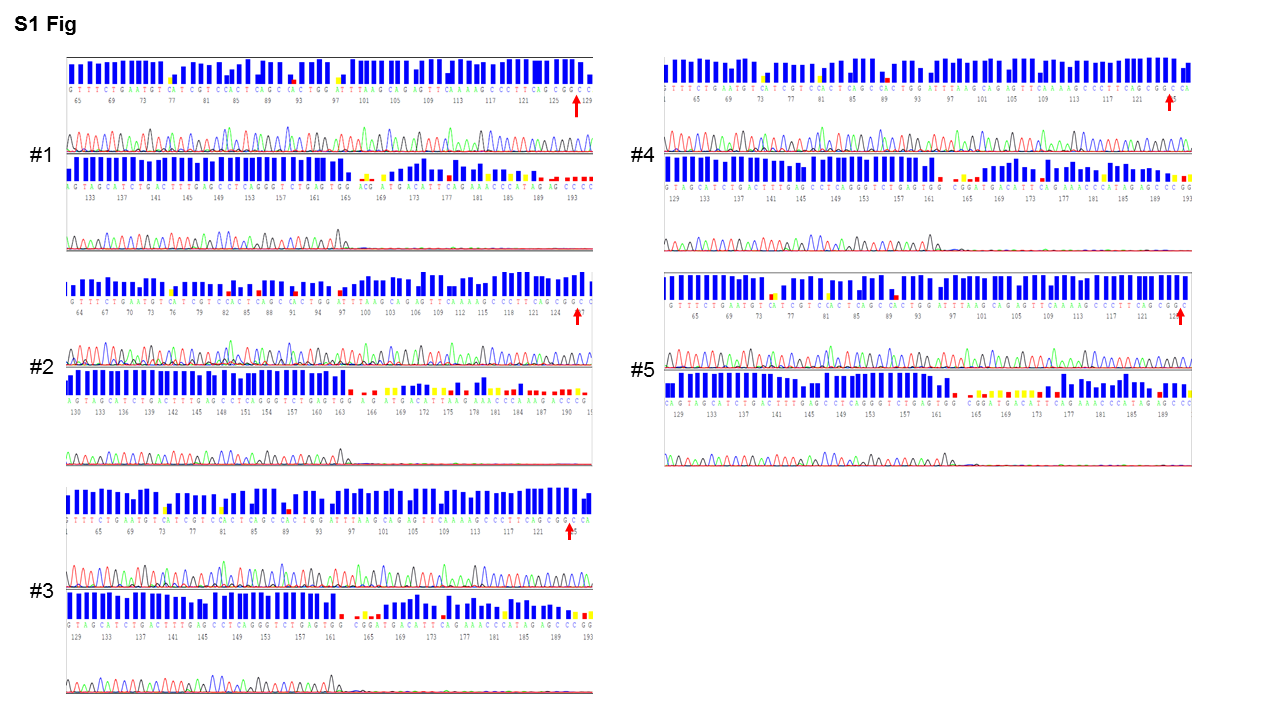

Supplement: S1 Fig — (TIF) [file pone.0207170.s003.tif]
